# UTR introns, antisense RNA and differentially spliced transcripts between *Plasmodium yoelii* subspecies

**DOI:** 10.1186/s12936-015-1081-9

**Published:** 2016-01-20

**Authors:** Jian Li, Baowei Cai, Yanwei Qi, Wenting Zhao, Jianwen Liu, Ruixue Xu, Qin Pang, Zhiyong Tao, Lingxian Hong, Shengfa Liu, Maarten Leerkes, Mariam Quiñones, Xin-zhuan Su

**Affiliations:** State Key Laboratory of Cellular Stress Biology, Innovation Center for Cell Signaling Network, School of Life Sciences, Xiamen University, Xiamen, Fujian 361005 People’s Republic of China; Laboratory of Malaria and Vector Research, National Institute of Allergy and Infectious Diseases, National Institutes of Health (NIH), Bethesda, MD 20892 USA; Department of Parasitology, Bengbu Medical College, 2600 Donghai Dadao Road, Bengbu, 233030 People’s Republic of China; Bioinformatics and Computational Biosciences Branch, National Institute of Allergy and Infectious Diseases, National Institutes of Health, Bethesda, MD 20892 USA

**Keywords:** Malaria, RNA-seq, Gene expression, Single nucleotide polymorphism (SNP), Alternative splicing

## Abstract

**Background:**

The rodent malaria parasite *Plasmodium yoelii* is an important animal model for studying host-parasite interaction and molecular basis of malaria pathogenesis. Although a draft genome of *P. yoeliiyoelii* YM is available, and RNA sequencing (RNA-seq) data for several rodent malaria species (RMP) were reported recently, variations in coding regions and structure of mRNA transcript are likely present between different parasite strains or subspecies. Sequencing of cDNA libraries from additional parasite strains/subspecies will help improve the gene models and genome annotation.

**Methods:**

Here two directional cDNA libraries from mixed blood stages of a subspecies of *P. yoelii* (*P. y. nigeriensis* NSM) with or without mefloquine (MQ) treatment were sequenced, and the sequence reads were compared to the genome and cDNA sequences of *P. y. yoelii* YM in public databases to investigate single nucleotide polymorphisms (SNPs) in coding regions, variations in intron–exon structure and differential splicing between *P. yoelii* subspecies, and variations in gene expression under MQ pressure.

**Results:**

Approximately 56 million of 100 bp paired-end reads were obtained, providing an average of ~225-fold coverage for the coding regions. Comparison of the sequence reads to the YM genome revealed introns in 5′ and 3′ untranslated regions (UTRs), altered intron/exon boundaries, alternative splicing, overlapping sense-antisense reads, and potentially new transcripts. Interestingly, comparison of the NSM RNA-seq reads obtained here with those of YM discovered differentially spliced introns; e.g., spliced introns in one subspecies but not the other. Alignment of the NSM cDNA sequences to the YM genome sequence also identified ~84,000 SNPs between the two parasites.

**Conclusion:**

The discoveries of UTR introns and differentially spliced introns between *P. yoelii* subspecies raise interesting questions on the potential role of these introns in regulating gene expression and evolution of malaria parasites.

**Electronic supplementary material:**

The online version of this article (doi:10.1186/s12936-015-1081-9) contains supplementary material, which is available to authorized users.

## Background

Malaria remains a major human infectious disease, killing hundreds of thousands of people each year [1]. The molecular basis of various disease symptoms or pathogenesis remains largely unknown due to difficulties in studying malaria infections in human hosts, including ethical concerns, genetic variations in human hosts and parasite strains, and co-infections with other pathogens. Because of better-controlled experimental environments, several rodent malaria parasites (RMP, i.e., *Plasmodium yoelii*, *Plasmodium berghei*, and *Plasmodium chabaudi*) have been established as animal models for studying molecular mechanisms of malaria pathogenesis, drug resistance, parasite development, and host-parasite interaction [2–5]. Although there are differences in disease mechanism between human and mouse malaria infections, they share many characteristics in disease symptoms and in host responses such as pro-inflammatory immune responses [6]. Among the RMPs, *P. yoelii* has been used as an animal model for vaccine development and for studying the genetic basis of parasite invasion and virulence [7–9]. Recently, various genetic markers [10–12] and genetic crosses have been reported for mapping important traits, such as red blood cell (RBC) invasion, parasite growth, and host cytokine/chemokine levels in *P. yoelii* infection [8, 13, 14] using clonal *P. yoelii* lines that exhibit wide variation in disease genotypes [9, 15, 16]. However, genetic mapping can only link chromosome segments containing various candidate genes to a phenotype. Identification of a causative gene generally requires further structural and functional confirmation of candidate genes, including experimental confirmation of individual gene structure (intron/exon boundary), expression, and function, which is usually time-consuming and labour-intensive. Systematic verification of predicted gene models and gene expression using cDNA sequences represents an efficient approach for gene model verification, which will greatly facilitate the genetic studies of the RMPs.

For *P. yoelii*, a draft genome of the *P. y. yoelii* 17XNL strain was published using Sanger dye-termination method more than 10 years ago [17]. Additionally, RNA sequencing (RNA-seq) was performed using RNA extracted from wild-type and *pypuf2*—sporozoites 14 days post-mosquito blood meal for comparison of gene expression level, but not for gene model verification [18]. Recently, RNA-seq data from three rodent malaria parasites were also reported, which greatly improved gene-model, gene expression, and subtelomeric multigene families [19]. However, errors and uncharacterized introns are still likely present in the RMP genomes. For example, errors in gene structure prediction such as missing introns, incorrect intron/exon boundaries, genes with alternatively spliced transcripts, and new transcripts missed by computer prediction were reported for the human malaria parasite *Plasmodium falciparum* even after several RNA-seq studies [20–25]. In this regard, additional RNA-seq data from different strains or subspecies of RMPs may reveal valuable information for further re-fining gene models and for detecting uniquely transcribed genes in specific parasite strains/subspecies. In addition, genome-wide sequencing of cDNA libraries from parasites with or without drug pressure may allow quantification and comparison of gene expression that could be linked to parasite response to a drug.

In this study, two cDNA libraries from a subspecies of *P. yoelii*, *P. y. nigeriensis* NSM (NSM), were sequenced with or without mefloquine (MQ) treatment. mRNA samples from mixed blood stages of the parasites were extracted, directional cDNA libraries were prepared, and RNA-seqs were performed to obtain large numbers of sequence reads. Comparison of the RNA-seq sequences to the assembled YM genome and recently published RMP cDNA sequences detected introns in 5′ and 3′ untranslated regions (UTRs), alternatively spliced transcripts, overlapping reads in opposite directions, and putative new gene transcripts. Differential/alternative splicing and SNPs between YM and NSM parasites, and variations in gene expression between NSM parasites with or without MQ pressure, were also observed. This study improves gene models in the *P. yoelii* genome and identified various UTR introns that may play an important role in regulation of gene expression.

## Methods

### Parasites and infection of mice

The NSM parasite was obtained from Malaria Research and Reference Reagent Resources [26]. The parasite is a mefloquine-resistant clone selected from *P. yoelii* NS that is a parasite line emerged from an isolate of *P. berghei* from Katanga, Belgian Congo, in the early 1970s [13, 27]. Ten inbred BALB/c mice were randomly divided into two groups (group 1 and group 2), and each mouse was injected *i.p.* with an inoculum containing 1 × 10^6^ infected red blood cells of NSM (iRBCs). Blood stage parasites were collected on day 4 after the injection. Mice from group 2 (NSM_2) were treated with mefloquine hydrochloride (40 mg/kg) for 3 h before parasite collection, whereas mice from group 1 (NSM_1) received no drug treatment. Female outbred ICR mice and inbred BALB/c, aged 6–8 weeks, used to maintain and grow the parasites, were purchased from Xiamen University Laboratory Animal Center and Shanghai Laboratory Animal Center, CAS (SLACCAS), respectively. The experimental procedures of mouse infection were performed according to protocols approved by the Laboratory Animal Management and Ethics Committee of Xiamen University (permit #XMULAC20120016) and by the Animal Care and Use Committee of the National Institute of Allergy and Infectious Diseases (NIAID), National Institutes of Health (permit #LMVR11E).

### RNA sample collection and preparation

RNA samples were prepared from mixed blood stages (~30 % ring, ~60 % trophozoite, ~9 % schizont, and ~1 % gametocyte) of the parasites using a Trizol RNA Extraction Kit (Life technologies, Carlsbad, CA, USA). Briefly, cells from 1 ml blood collected in 10 ml 0.15 % sodium citrate/PBS buffer were pelleted by centrifugation (2000 rpm, 5 min), re-suspended in 10 ml of PBS, and passed through two NWF filters (Zhixing Bio, Bengbu, China) to remove the host white blood cells [28]. The flow-through cell suspension was centrifuged at 3000 rpm for 3 min. The cell pellet was washed 2× in PBS, re-suspended in 10 ml PBS, and lysed with 0.05 % saponin on ice. The sample was centrifuged at 6000 rpm for 3 min at 4 °C, washed 2X in PBS, and dissolved in Trizol for RNA extraction following manufacturer’s instructions. RNA quality and purity were first monitored using 1 % agarose gels (Additional file 1a) and IMPLEN NanoPhotometer (Westlake Village, CA, USA). RNA concentration was determined using Qubit RNA Assay Kit in a Qubit^®^ 2.0 Flurometer (Life Technologies, Frederick, MD, USA). RNA integrity was further assessed using the RNA Nano 6000 Assay Kit and the Bioanalyzer 2100 system (Agilent Technologies, Columbia, MD, USA).

### Strand-specific library preparation and sequencing

A total of 3 μg RNA per sample was used in sequencing library construction using NEBNext UltraTM Directional RNA Library Prep Kit for Illumina^®^ (NEB, Ipswich, MA, USA) following manufacturer’s recommendations. Briefly, mRNA was purified from total RNA using polyT oligo magnetic beads. Fragmentation was carried out using divalent cations under elevated temperature in the NEBNext First Strand Synthesis Reaction Buffer. First strand cDNA was synthesized using random hexamer primers and M-MuLV reverse transcriptase (RNaseH-). The second strand cDNA was synthesized using DNA polymerase I and RNase H. For strand-specific sequencing, dTTP was replaced with dUTP during cDNA synthesis. After converting overhanging ends into blunt ends and adenylation of 3′ ends of DNA fragments, NEBNext adaptors with hairpin loop structure were ligated to the cDNAs, and fragments of 150–200 bp were purified using AMPure XP system (Beckman Coulter, Sykesville, MD, USA). The samples were then treated with 3 μl USER Enzyme (Uracil-Specific Excision Reagent, NEB) at 37 °C for 15 min followed by 5 min at 95 °C before PCR amplification. The amplification was performed using Phusion high-fidelity DNA polymerase, Universal PCR primers, and Index (X) Primer. The PCR products were purified using an AMPure XP system. The quality of the libraries was assessed using Agilent Bioanalyzer 2100. Index-coded samples were clustered in a cBot Cluster Generation System using TruSeq PE Cluster Kit v3-cBot-HS (Illumina, San Diego, CA, USA) according to the manufacturer’s instructions. After clustering, the libraries were sequenced in an Illumina Hiseq 2000 platform and 100 bp paired-end reads were generated.

### Sequence reads cleanup and mapping to genome

A flow chart of sequence reads cleanup and data analysis is presented in Additional file 1b. Briefly, raw sequence reads of fastq format were first processed using custom perl scripts. Clean reads were obtained by removing reads containing adapter, reads containing poly-N, and those with low quality scores (<Q20). At the same time, percentages of reads with Q20, Q30, and GC content were calculated. All the downstream analyses were based on the clean data with a quality score ≥Q20. The analysed RNA-Seq data for this study has been submitted to the Sequence Read Archive (SRA) at NCBI and can be viewed under accession number SRP066796 [29].

Paired-end clean reads were mapped to the YM reference genome at the Sanger website [30] and compared with the gene models predicted from the YM genome sequences after building the index of the reference genome using TopHat v2.0.9 11 and Bowtie v2.0.6 [31–34] implemented with mismatch = 3 and default parameters, initially. For the detection of new splicing variants, the data were re-run using TopHat v2.0.11 and Bowtie2 v2.2.2 by applying parameters of mismatch = 2, read edit distance = 3, and max intron size = 1000 to filter split reads >1 kb and non-specific reads. TopHat was selected as the mapping tool because it can generate a database of splice junctions based on the gene model annotation file.

### Read counts and estimation of gene expression levels

HTSeq v0.5.4p3 was used to count the number of reads mapped to each gene for quantification of gene expression level, expressed as reads per kilobase per million or RPKM [35]. Gene expression analysis was performed using a DESeq R package [36]. DESeq provided statistical routines for determining differential expression using a model based on the negative binomial distribution. The resulting *P*-values were adjusted using the Benjamini and Hochberg’s approach for controlling the false discovery rate (FDR), and genes with an adjusted *P* value <0.05 were considered differentially expressed.

### Detection of new transcripts and events of alternative transcripts

The Cufflinks v2.1.2 Reference Annotation Based Transcript (RABT) assembly method was used to construct and identify both known and novel transcripts from TopHat alignment results [33, 37]. Alternative splicing (AS) events were detected initially using the software Asprofile v1.0 [38]. In addition to AS, incorrect intron/exon boundaries, overlapping sense-antisense transcripts, and putative new transcripts missed by computer prediction were also characterized. Putative ‘introns’ with split reads larger than 1 kb (unlikely true for this parasite) and those having fewer than five reads coverage were considered artifacts and were removed before further analysis. The mapped reads were visually inspected in Integrative Genomics Viewer (IGV) [39] to ensure the accuracy of the detected mismatches to the YM gene models. TransDecoder software [40] was used to identify coding regions within transcripts generated by Cufflinks.

### Detection of SNPs and indels

Because NSM and YM have quite different genomes [10, 12], Samtools v0.1.18 and Picard-tools v1.96 were used to sort and mark duplicate reads [41], and then performed SNP and indel calling using GATK2 [42]. Only the putative SNPs or indels with 10 or more reads coverage were recorded in order to reduce the numbers of false positive SNPs arising from sequencing and alignment errors.

### Experimental confirmation of selected SNPs and AS events

To further confirm the accuracy of the RNA-seq data in predicting alternative spliced introns and SNP detection, PCR primers flanking selected SNPs and AS events were designed, and PCR products were obtained from both genomic and cDNA of the NSM and YM parasites. The PCR products were either sequenced directly using the Sanger dye terminator method for SNP confirmation or separated on agarose gels for size polymorphisms to detect spliced introns. PCR reactions in 15 μl were performed under conditions described previously [10], with a cycling programme of 94 °C for 2 min for initial denaturation; 94 °C, for 20 s; 55–50 °C, for 20 s; 60 °C, for 30 s for 35 cycles; and a final extension at 60 °C for 2 min.

## Results

### Directional RNA-seq

To study gene expression and improve gene models, two RNA samples of mixed blood stages from the NSM parasite were prepared, directional cDNA libraries were constructed, and RNA-seqs were performed by a commercial company (Novogene, Beijing, China). One of the two RNA samples was from parasites treated with MQ (40 mg/kg) for three h, and the other had no drug treatment. Approximately 14.5 million raw reads and approximately 14 million clean reads were obtained after removing adapters and low quality reads from run (2 runs per samples), generating a total of ~56 millions of 100 bp paired-end read (Additional file 2). The reads provided ~225-fold averaged coverage of the coding regions of the parasite genome for each sample (assuming 50 % coding sequences and 25 mb genome size). Among the raw reads, ~99 % had a Phred quality score (QS) of 20 or higher and ~96 % of the reads had QS30 or higher, with an estimated error rate of ~0.02 % or one error per 10,000 bp. As expected, the GC content was approximately 28–29 % for all the sequences, consistent with a reported genome consisting of ~77 % AT [43]. These results suggest sequencing reads with excellent quality from the RNA samples.

### Mapping sequence reads to chromosomes

The clean reads from each sample were mapped to the assembled chromosomes of *P. y. yoelii* YM parasite available at the Sanger website [30] using Tophat2 and ~72–73 % of the reads were assigned to chromosomes initially. Inspection of the mapped reads in IGV showed that some split-reads spanned more than 1 kb sequence, which is unlikely to be true for malaria parasites. Additional ‘filters’ (mismatch = 2, read edit distance = 3, and max intron size = 1000) were applied to remove split-reads with gaps ≥1 kb and low quality non-specific mapped reads. The new analyses mapped ~66 % reads to the chromosomes, with ~7 % split-reads (with gaps) and ~1 % reads having multiple hits that could represent reads from multi-gene families (Table 1). The mapped reads were distributed quite evenly across all of the 14 parasite chromosomes, with log2 median read density ~15 per kb for each strand (Additional file 3). Approximately 88–90 % of the mapped reads were located at exons and ~10–12 % at intergenic regions or 5′/3′ UTRs, with relatively even distribution to the ‘+’ and ‘−’ DNA strands (Additional file 3 and Table 1). The numbers of reads mapped to chromosomes was positively correlated with the chromosome sizes, with a linear relationship for both of the parasite samples (Additional file 4).

**Table 1:** Statistics of sequence reads mapped to chromosomes

Sample name	No. reads (NSM_1)	% reads (NSM_1)	No. reads (NSM_2)	% reads (NSM_2)
Total reads	2,808,0144		2,825,3076	
Total mapped	1,863,0250	66.3	1,862,9614	65.9
Multiple mapped	28,1316	1.0	26,3365	0.9
Uniquely mapped	1,809,6655	64.4	1,812,8822	64.2
Read-1	890,8816	31.7	891,7725	31.6
Read-2	918,7839	32.7	921,1097	32.6
Reads map to ‘ + ’	904,9370	32.2	906,2606	32.1
Reads map to ‘-’	904,7285	32.2	906,6216	32.1
Non-splice reads	1,611,0463	57.4	1,604,6313	56.8
Splice reads	198,6192	7.1	208,2509	7.4

Multiple mapped, number of reads mapped to multiple sites; uniquely mapped, number of reads mapped to one site only; Read-1 and Read-2, reads from the two separate lanes; Reads mapped to ‘+’ or ‘−’, mapped to ‘+’ or ‘−’ strands of DNA; Splice reads, number of reads mapped to splice sites

### Estimates of gene expression levels

To evaluate the gene expression levels, RPKM [35] was calculated for each predicted gene. Approximately 60 % of the genes (or ~4000 genes) were expressed at relatively high levels with RPKM ≥15; ~20 % of the genes were not expressed or were expressed at low levels (PRKM = 0–3) (Table 2 and Additional file 5). As expected, the 500 most highly expressed (PRKM >280) genes were those encoding ribosomal proteins, putative antigens, and other essential genes of parasite metabolism, whereas the 500 genes expressed at the lowest levels (or not expressed) were largely those encoding YIR proteins, fam-a proteins, or some stage-specific expressed genes (Additional file 5). Other noticeable genes not detected included apicoplast encoded ribosomal proteins, RNA polymerase B (rpoB), RNA polymerase beta (rpoC), and RNA polymerase D (rpoD), and sporozoite-specific thrombospondin-related anonymous protein (TRAP), some of which may be expressed in liver and/or mosquito stages. Because apicoplast transcripts typically do not have polyA tails, the lack of sequences from apicoplast-encoded genes could be due to the polyT purification step used to capture full-length mRNAs with polyA tails.

**Table 2:** The number and percentages of genes with different expression levels as estimated using reads per kilobase pair per million reads (RPKM)

RPKM range	No. genes (NSM_1)	% genes	No. genes (NSM_2)	% genes
0–1	874	12.7	915	12.3
1–3	628	9.1	609	8.8
3–15	1305	18.9	1248	18.1
15–60	1917	27.8	1856	26.9
>60	2167	31.5	2263	32.8

**Fig. 1:**
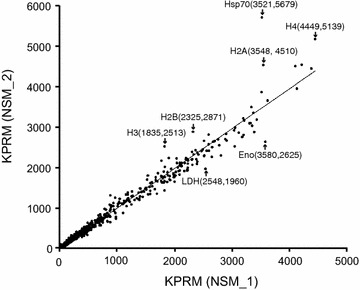
Correlation plot of gene expression levels (reads per kilobase per million or RPKM) between NSM_1 and NSM_2. Only genes with RPKM ≥10 and ≤6000 in NSM_1 were plotted; seven genes with PRKM ≥6000 were excluded in order to show genes with lower expression. *Hsp70* heat shock protein 70, *H4* histone H4, *H2A* histone H2A, *H2B* histone H2B, *H3* histone H3, *Eno* enolase, *Ldh* l-lactate dehydrogenase

Because the NSM parasite was MQ selected, treatment of the parasite with MQ might trigger the expression of genes playing a role MQ response. However, the gene expression levels between the two samples, with or without MQ treatment, were also very similar. Plots of gene expression levels (10 ≤ RPKM values ≤ 6000; total 4467 genes) showed very good correlation in gene expression between the samples (Fig. 1), suggesting consistency in mRNA preparation and DNA sequencing. A few examples of genes that were obviously expressed at higher level in the MQ-treated sample (NSM_2), included a gene encoding heat shock protein 70 (HSP70) and four genes encoding histone proteins (Fig. 1). Other genes such as the genes encoding a putative enolase (ENO) and an l-lactate dehydrogenase (LDH), respectively, were expressed at lower level in the MQ-treated parasite. The functional roles of the differentially expressed genes in MQ-response are currently unknown and require further investigation. The increase in HSP70 expression could represent general stress response to the pressure from the drug.

### Alternatively and differentially spliced introns between two *Plasmodium yoelii* subspecies

The collection of large numbers of cDNA reads allows construction of gene models and comparison of the gene structures with those predicted based on genome sequences. First, ASprofile and Cufflinks software [37, 38] were used to extract and identify alternative splicing (AS) events observed in the cDNA gene models after comparison with those predicted from YM genome sequence. Next, ‘filters’ were applied to remove potential artifacts, including genes with <40 RPKM (to ensure sufficient read coverage at splicing sites) and split reads with gaps >1 kb. Finally, the genes with gene models different from those predicted in the YM genome (intron–exon structure, new intron, alternative splicing, etc.) were visually inspected in IGV after alignment of the reads from NSM_1 and NSM_2 to the updated YM genome sequence and then compared with the recently re-annotated genes using RMP cDNA sequences [19].

**Fig. 2:**
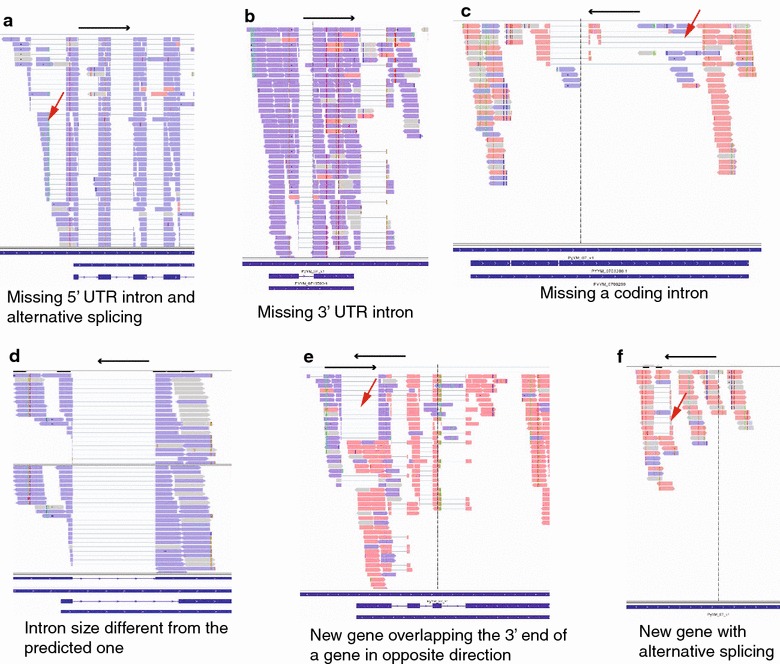
Types of mismatches in gene models detected after alignment of directional cDNA sequences to the YM genome sequences. **a** Alternatively-spliced introns at 5′ UTR; **b** an intron at the 3′ UTR; **c** an intron within coding region; **d** an intron smaller than the one predicted, possibly an alternatively-spliced intron, too; **e** overlapping transcripts in two neighboring genes; **f** a putative new transcript with an intron. The figures are images from Integrative Genomics Viewer (IGV). *Purple* sense reads; *pink* antisense reads. The *blue bars* are predicted gene models from the *Plasmodium y. yoelii* YM genome

The alignment of NSM cDNA reads to the YM genome showed high accuracy in the YM predicted gene models; however, various introns and putative transcripts not reported previously were also detected, including 5′ and 3′ UTR introns (Fig. 2a, b), new coding introns (Fig. 2c), introns with sizes not matching those predicted (Fig. 2d), overlapping transcripts with introns (Fig. 2e), and putative new transcripts (Fig. 2f). The new introns identified from the NSM sequences were compared with those reported recently from the YM parasite [19], and the results showed that not all the new introns detected from YM and NSM were the same (Additional file 6). The YM RNA reads from Otto et al. [19] and the NSM reads were then aligned to the YM genome sequence, and the aligned sequence reads were visually inspected in IGV together. The results of this analysis are summarized in Table 3 and Additional file 6. Comparisons of RNA-seq reads from both NSM and YM with the YM genome detected 625 and 629 ‘events’ such as new UTR and coding introns or incorrect intron size in NSM and YM, respectively, in 652 genes. Among the events, the majorities (50.4 % for NSM and 45.5 % for YM) are putative 5′ or 3′ UTR introns. However, there were also genes with split-reads that did not have the eukaryotic intron boundary GT-AG, and these events were likely size polymorphisms. The results suggest an excellent job of the YM genome annotation that produces very few errors in intron/exon structure in the coding regions. The detection of relatively large numbers of 5′ and 3′ UTR introns indicate that these UTR introns could play an important role in gene expression regulation.

**Table 3:** Transcriptional events such as introns or overlapping transcripts not predicted in YM gene models

Events	NSM	YM	NSM or YM	NSM and YM	NSM only	YM only	% in Both
5’ UTR intron	245	227	254	218	27	9	85.8 %
3’ UTR intron	70	59	74	55	15	4	74.3 %
Sense/antisense overlapping	29	26	32	23	6	3	71.9 %
Alternative splicing	52	47	55	44	8	3	80.0 %
Internal/coding intron	60	37	61	36	24	1	59.0 %
Transcript extension^a^	52	63	64	51	1	12	79.7 %
Incorrect intron size	12	13	13	12	0	1	92.3 %
Others^b^	105	157	164	98	7	59	59.8 %
Total	625	629	717	537	88	92	74.9 %

The YM data were from [19]

^a^Indicates possible longer coding, or transcript extension on 5′ or 3′

^b^Including overlapping transcript, putative new transcripts, etc

**Fig. 3:**
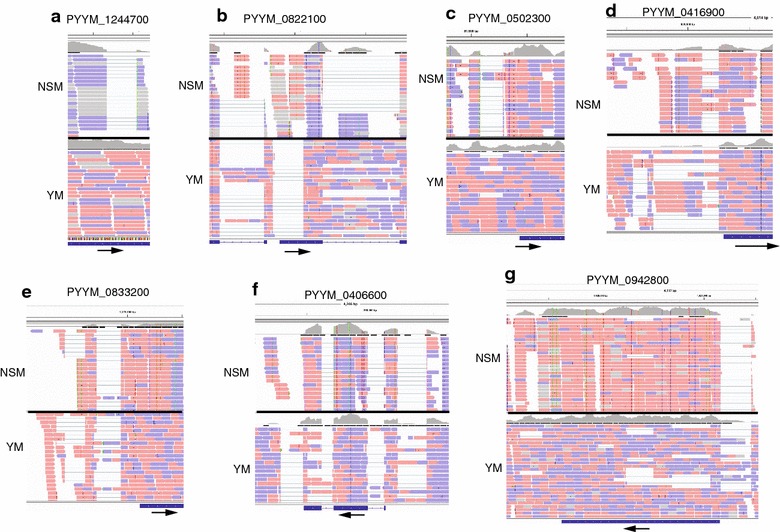
Examples of differentially (alternatively) spliced introns between *Plasmodium y. yoelii* YM and *P. y. nigeriensis* NSM. cDNA reads from YM (Otto et al. [19]) and NSM were compared using IGV. Gene IDs are on top of each panel. Reads in pink are sense, and those in* purple* are antisense. **a** A coding intron present in NSM, but not in YM; **b** an intron in YM, but not in NSM; **c**, **d** 5′ UTR introns present in both YM and NSM, but the proportions of reads with spliced forms are different. For PYYM_0502300, most of the reads from NSM are spliced, but not those of YM (**c**). **e** additional sliced forms of 5′ UTR introns are present in YM, but not NSM; **f** an additional alternatively spliced coding intron in YM, but not NSM. YM may also have an extra 3′ UTR intron; **g** one intron in NSM, but multiple introns in antisense transcripts in YM, which may generate a large number of variants (>10)

Comparison of the aligned cDNA reads of YM and NSM showed that ~75 % of the new events were present in both parasites (Table 3 and Additional file 6). Interesting examples of variation in intron splicing events between the two parasite subspecies included: (1) Introns that were present in one subspecies (NSM or YM) but not in the other (Fig. 3a, b), which might explain some of the differences in the gene lists; (2) presence of reads with or without an intron in both parasites, but the percentages of reads with an intron was different (Fig. 3b, c): for example, the majority of reads from NSM had a spliced 5′ UTR intron for the PYYM_0502300 gene, but the majority of the reads from YM did not have the intron spliced (Fig. 3c); (3) presence of different forms and numbers of alternatively spliced introns (Fig. 3d–f): for example, NSM had two 5′ UTR intron forms for PYYM_0416900, whereas YM had four alternatively sliced forms (Fig. 3d); and (4) multiple alternatively spliced introns at different positions in antisense transcripts (Fig. 3g): for gene PYYM_0942800, the transcripts with different intron-spliced forms were all antisense in YM.

These observations suggest that YM and NSM not only differ in DNA sequences, but also have different intron splicing patterns. In theory, these differentially spliced introns between *P. yoelii* subspecies could be caused by SNPs at intron splicing sites. To investigate this possibility, the splicing site sequences in 58 genes having introns that were present only in YM or NSM were compared; only three genes had multiple introns, with one of the introns having a substitution at the intron splicing sites that might lead to disruption of intron splicing (Additional file 6). The differentially spliced introns observed between YM and NSM can be due to lower read coverage for one of the subspecies or by mechanisms of unknown processes. The alternatively/differentially spliced introns between parasite strains/subspecies will generate a large numbers of alternatively expressed genes in parasite populations.

### Detection of SNPs between YM and NSM parasites

The cDNA sequences from the two NSM samples were compared with the YM genomic sequence to detect potential SNPs between NSM and YM parasites. Samtools and Picard-tools were used to sort and remove duplicate reads, and then Genome Analysis Toolkit 2 (GATK2) was applied to detect SNPs and indels. From the aligned sequences, reads with alleles different from the reference YM sequence were recorded. Using criteria of at least 10-read coverage and >75 % calling for an alternative allele to filter potentially low quality SNP calls, 84,431 SNPs between NSM_1/NSM_2 and YM were detected (Additional file 7), but no SNP between NSM_1 and NSM_2 met the criteria (as expected). The large number of SNPs detected between YM and NSM are consistent with the report of highly diverse genomes between YM and N67 parasites (both N67 and NSM were derived from *P. yoelii* NS) [12]. No indel with 10 or more reads coverage was detected between NSM_1 and NSM_2.

### Genes with antisense transcripts

**Fig. 4:**
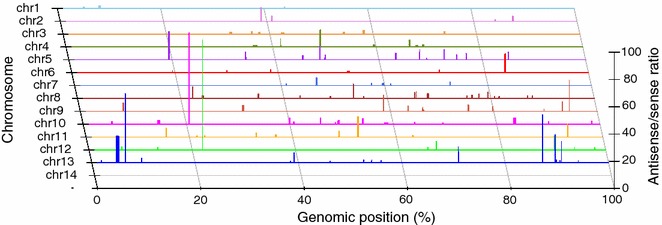
Distribution of genes with antisense/sense (AS:S) ratio ≥1 across the 14 parasite chromosomes. Each horizontal line represents a chromosome. The relative positions of the genes with antisense reads ≥sense reads on the chromosomes are as marked, with longer vertical lines representing higher antisense/sense ratios

Although the antisense-sense ratios (AS:S) were reported to be unevenly distributed across the mouse genome [44], exons with high AS:S ratios in the *P. falciparum* genome were found to be randomly distributed across the 14 parasite chromosomes [21]. The construction of directional *P. yoelii* cDNA libraries also allows detection of antisense and sense transcripts across the whole genome. Similar to those of *P. falciparum*, analysis of the AS:S ratios for predicted exons across the 14 chromosomes of the *P. yoelii* genome showed that exons/genes with high AS:S ratios were also randomly distributed in the *P. yoelii* genome (Fig. 4 and Additional file 8). There were 176 exons with at least five reads or more in both directions per exon and with higher antisense read coverage than that of sense (Additional file 8), compared with 312 exons in *P. falciparum* reported previously [21]. The higher number of exons with antisense reads detected in *P. falciparum* could be due to the fact that more cDNA libraries from different stages (including sexual stages) were sequenced in the previous study. Except for genes encoding conserved *Plasmodium* proteins, there was no clear enrichment of GO-terms among the genes expressing high levels of antisense transcripts.

### Confirmation of selected SNPs and alternatively/differentially spliced introns

**Fig. 5:**
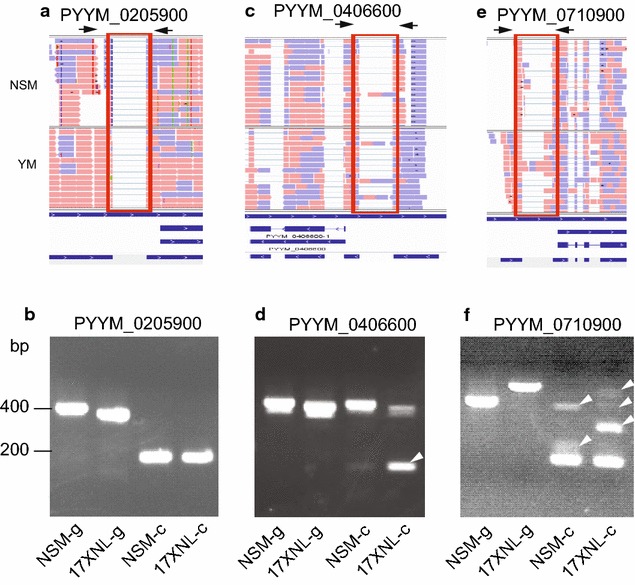
Verification of differentially spliced introns between *Plasmodium y. nigeriensis* NSM and *Plasmodium y. yoelii* 17XNL. DNA and mRNA samples were prepared from NSM and 17XNL parasites; 17XNL was used because YM parasite was not available in the laboratory in China. **a**, **b** RNA-seq read alignments showing a predicted intron and primer positions (*arrows*) from the PYYM_0205900 gene that has the same spliced intron in both NSM and YM (**a**) and amplification products using primers 5′-TGTCCATCAAATAATAAAGCTAAAATATATTCCTCTCA-3′ and 5′-TATAGTTAGATGTGTTTAATATTTAAGG-3′. Parasite names with ‘g’ indicate amplification products from genomic DNA, and those with ‘c’ were from cDNA. The results showed no DNA band in the cDNA preparations, suggesting lack of DNA contamination in the cDNAs. **c**, **d** RNA-seq alignments (**c**) and amplification products (**d**) from gene PYYM_0406600 using primers 5′-GTAAGAAATATACAACAATACTATTCCTTGGCAA-3′and 5′-CTCTCCCATTTTTAGGTATAAAAAATAACTAAATATG-3′, showing a much stronger sliced product (*arrowhead*) in 17XNL than in NSM. **e**, **f** RNA-seq alignments (**e**) and amplification products from gene PYYM_0710900 (**f**) showing differential spliced bands (*arrowheads*) between NSM and 17XNL parasites (primers: 5′-GATTTCTATTAGCTTTGTGAAGTC-3′ and 5′- TGTAATATATTATCGAAAGACGTG-3′). In addition to differential splicing, size polymorphism in genomic DNA between NSM and 17XNL was also detected

To confirm alternatively and differentially spliced introns, PCR primers were synthesized to amplify introns from three different genes (Fig. 5). Genomic and cDNA from both NSM and *Plasmodium yoelii yoelii* 17XNL (17XNL) parasites were amplified (The YM parasite was not available at the Xiamen laboratory and, therefore, the genetically closely related 17XNL parasite was used [15]), and the PCR products were separated on 1–2 % agarose gels. Comparison of products from genomic DNA allowed detection of size polymorphism between the two parasites, whereas comparison of products from cDNA and genomic DNA sequences from the same parasite will detect splicing events. Based on RNA-seq alignments, gene PYYM_0205900 had a predicted 5′ UTR intron in both NSM and YM parasites (Fig. 5a), and PCR products confirmed the predictions with products of expected sizes (188 pb after splicing, Fig. 5b). The lack of DNA band in the cDNA products also suggested no (or undetectable) DNA contamination in the cDNA samples. Gene PYYM_0406600 had a predicted spliced product (142 bp), but the spliced band in 17XNL was much stronger than that of NSM (YM should have band intensity similar to NSM according to RNA-seq reads). The results also suggested ‘uneven’ intron splicing between the NSM and 17XNL parasites (Fig. 5c, d). Finally, gene PYY_0710900 had different forms of spliced 5′ UTR introns in RNA-seq alignments (Fig. 5e), and the sizes of PCR products generally agreed with the RNA-seq predictions, although PCR appeared to detect some additional low level spliced forms (Fig. 5f); NSM-c produced three bands, whereas 17XNL-c had four bands, with at least three bands having different sizes between the two parasites, These results suggest multiple differentially spliced intron forms within and between parasites.

To investigate whether the ‘introns’ without GT-AG sites were true introns or simply size polymorphisms, PCR primers flanking predicted ‘introns’ that had split-reads with or without the conserved GT-AG intron-splicing boundary in 23 genes were synthesized to amplify genomic and cDNA from the NSM parasites (Additional file 9). None of the six putative introns without GT-AG intron splicing boundary had a cDNA band smaller than the genomic band, suggesting that they were not true introns, whereas 13 of the 17 (76.5 %) with introns having GT-AG sites had at least one cDNA band with size smaller than the genomics bands (Additional file 9).

In addition, 26 SNPs (most of which had read coverage below 50) from the 84,431 putative SNPs were randomly selected, and DNA sequences spanning the SNPs from NSM and YM were amplified and directly sequenced using the Sanger dye terminator method. Among the 26 putative SNPs, 25 (96 %) were confirmed, except for one (PYYM_1355300) due to sequencing failure (Additional file 10). The results suggest high accuracy in the SNP detection.

## Discussion

This study reports genome-wide sequencing and characterization of directional cDNA libraries from *P. yoeliinigeriensis* parasite, generating ~56 million high-quality paired-end sequence reads. Approximately ~66 % of the reads were mapped to the assembled YM genome sequence, providing good coverage across the 14 parasite chromosomes. This study also shows that more than 50 % of the genes were transcribed at relatively high levels (RPKM ≥ 15) in the NSM parasite, and ~11–13 % of the genes were either not expressed or expressed at low levels in blood stages. The genes with no RNA detected could be expressed in liver or mosquito stages that were not examined in this study. The relatively high proportion of genes being expressed is not surprising because the cDNA libraries were prepared from mRNAs of mixed stages.

The relatively large numbers of cDNA sequences obtained in this study allowed comparison of gene structures and detection of gene model prediction errors. The results show that gene structures/models for this *P. yoelii* subspecies (NSM) are very similar to those of YM parasite. Consequently, the majority of new introns identified were at UTR regions, particularly 5′ UTRs. The observations of UTR introns raise an interesting question of whether the alternatively spliced non-coding introns play a role in regulating gene expression in malaria parasites. Indeed, introns in UTRs have been found in many organisms; approximately 35 % of 5′ UTRs and ~16 % of 3′ UTRs of human genes contain introns [45]. Recent studies have shown that genes with regulatory functions are more likely to have 5′ UTR introns, and human 5′ UTR introns can enhance the expression of some genes in a length-dependent manner [46]. In *Arabidopsis thaliana,* the density of introns in 5′ UTR was found to be much higher than in 3′ UTRs (~threefold), and the presence of a long intron in the 5′ UTR (i.e., intron not spliced out) in the EF1α-A3 gene could enhance the expression of the gene [47]. 5′ UTR introns can also influence how the mRNAs are exported from the nucleus; mRNAs with 5′ UTR introns are generally exported by the canonical transcription export (TREX) pathway, whereas those without 5′ UTR introns are exported through an alternative mRNA export (ALREX) pathway [45]. Similar to those found in *Arabidopsis thaliana,* approximately three-fold more 5′ UTR introns than 3′ UTR introns were observed in *P. yoelii*, which could partly reflect generally longer 5′ UTRs than 3′ UTRs in genes of malaria parasites. Among the genes with 5′ UTR introns include those encoding ribosomal proteins, DNA/RNA binding proteins, transcription factors, and heat shock proteins (Additional file 6). Therefore, in addition to the well-known mechanisms such as transcription factors, promoters, silencers, enhancers, epigenetic regulators, and antisense transcripts, introns in non-protein coding regions may also play an important role in regulation of gene expression in malaria parasites. Further functional investigations are necessary to provide experimental evidence to support this claim.

Another interesting observation of this study was differentially (different proportion) spliced introns in some genes between YM and NSM parasites. These differences in intron splicing between the two parasite subspecies will likely contribute to differences in parasite biology and/or disease phenotypes, suggesting that genetic investigations such as linkage or association studies may need to consider the differences in intron splicing. The causes of these splicing differences are unknown, but the majority of them are not due to mutations at intron splicing sites between the two parasites, suggesting the presence of unknown mechanisms of intron splicing and gene expression regulation in malaria parasites.

Approximately half of the genes in the genome were not included in the analysis of alternatively spliced events due to low or lack of expression because only genes with RPKM ≥ 40 were analysed. The actual numbers of genes with various AS events could be much higher than those observed. Nonetheless, this study still detected many alternatively spliced events that improved gene models and genome annotation of the *P. yoelii* parasite. The main goals of this study were to detect expressed genes in blood stages and to verify intron/exon boundaries in the predicted gene models. Many transcripts that were not detected in this study, including those only expressed in liver and mosquito stages, can be evaluated if RNA samples are collected from different developmental stages.

Sequencing cDNAs from a parasite evolutionarily different from the YM parasite also allows detection of a large number of SNPs (~84,000), which is consistent with the previous report of a high level of diversity between YM and *P. y. nigeriensis* N67 [12]. Both N67 and NSM were derived from NS, so the genomes of NS, NSM, and N67 are essentially the same [12, 27]. To confirm the accuracy of the SNPs detected in this study, 26 putative SNPs with relatively low read coverage were randomly selected; DNA segments containing the SNPs were amplified; and the PCR products were sequenced directly. Except for one sequencing failure, all the 25 putative SNPs from RNA-seq were confirmed after PCR amplification and direct product sequencing using Sanger’s method. The results provide strong confidence in the SNPs detected in this study, and these SNPs will be useful for genetic studies of the *P. yoelii* parasites.

Examples of alternatively and differentially spliced introns within the same and between different parasites were also PCR amplified to support the observations of differentially spliced introns between the two parasite subspecies, although a variety of factors may contribute to the differences observed, including sequencing depth, variations in library construction, and differences in the parasite stages when RNA samples were prepared. The mechanism and functional significance of alternative and differentially sliced introns between parasite strains or subspecies will require further investigations.

PCR amplification of putative introns with or without the GT-AG intron splicing boundaries suggests that *P. y*. *nigeriensis* parasite also follows eukaryotic intron splicing mechanism requiring the GT-AG intron boundary for intron splicing. All six putative introns (or regions with split reads) without GT-AG sites turned out to be false, showing no spliced PCR band, whereas 13 of the 17 genes with introns having GT-AG sites had bands smaller than those from genomic DNA, suggesting the presence of introns. The four genes with putative introns that had GT-AG boundary but did not produce a smaller PCR band could be due to low-level expression of the alternatively spliced intron, or the introns were only spliced in YM (not tested here), but not in NSM and 17XNL.

## Conclusion

Although the data were obtained from mixed blood stages of the parasites, and many genes expressed in mosquito and the liver stages could not be characterized here, this study provides important information on gene structure, expression, and genome polymorphism between *P. yoelii* parasites. The detection of a large number of UTR introns should raise attention to the potential regulatory roles of these largely ignored introns and the molecular mechanisms of gene expression regulation by UTR introns. Analysis of genetic variations should consider the large numbers of alternatively spliced transcripts in a parasite and the differentially-spliced forms between parasite strains or subspecies.

## Additional files

10.1186/s12936-015-1081-9 Evaluation of RNA quality from the two NSM parasite samples in agarose gel (**a**), and a flow chart of data processing and analysis (**b**).
10.1186/s12936-015-1081-9 Sequence reads, read quality, and error rates.
10.1186/s12936-015-1081-9 Distribution of mapped sequence reads per kb across the 14 parasite chromosomes, mitochondrial (Mt), and apicoplast (Ap) genomes. The green and orange colors indicate ‘+’ and ‘-’ strand coverage, respectively. The chromosomes are as labeled. Only data from NSM_1 are shown. Bin contains sequences not assigned to chromosomes, likely due to gaps in the current assembled genome.
10.1186/s12936-015-1081-9 Correlation of chromosome length in megabase (Mb) and the numbers of mapped reads. The chromosomes are as labeled. Only data from NSM_1 are shown. The numbers above or below the orange dots indicate chromosomes; Mt, mitochondrial genome; Ap, apicoplast genome; bin, unassigned sequences.
10.1186/s12936-015-1081-9 Gene expression levels expressed as reads per kilobase per million reads (RPKM) in the NSM clones.
10.1186/s12936-015-1081-9 New genes or genes with structures that do not match the predicted gene models in the *Plasmodium yoelii* YM genome sequence.
10.1186/s12936-015-1081-9 Putative single nucleotide polymorphisms (SNPs) between *Plasmodium y. nigeriensis* NSM and *Plasmodium y. yoelii* YM.
10.1186/s12936-015-1081-9 *Plasmodium yoelii* exons/genes with high antisense over sense read coverage.
10.1186/s12936-015-1081-9 PCR verification of putative introns or size polymorphisms with or without GT-AG sites.
10.1186/s12936-015-1081-9 Confirmation of single nucleotide polymorphisms (SNPs) detected between *Plasmodium y. yoelii* YM and *P. y. nigeriensis* NSM.

## Abbreviations

AS:S: antisense:sense
bp: base pairs
cDNA: complementary DNA
RPKM: reads per kilobase per million reads
UTR: untranslated region
RBC: red blood cells
YM: *Plasmodium y. yoelii* YM
NSM: *Plasmodium y. nigeriensis* NSM
SNPs: single nucleotide polymorphisms
QS: Phred quality score
PCR: polymerase chain reaction
TREX: transcription export pathway
ALREX: alternative mRNA export pathway

## References

[1] WHO: Worldmalaria report 2012. World Health Organization. 2014. http://www.who.int/malaria/publications/world_malaria_report_2014/en/. Accessed 15 Jan 2016.

[2] Stephens R, Culleton RL, Lamb TJ (2012). The contribution of *Plasmodium chabaudi* to our understanding of malaria. Trends Parasitol.

[3] Carlton JM, Hayton K, Cravo PV, Walliker D (2001). Of mice and malaria mutants: unravelling the genetics of drug resistance using rodent malaria models. Trends Parasitol.

[4] Janse CJ, Kroeze H, van Wigcheren A, Mededovic S, Fonager J, Franke-Fayard B (2011). A genotype and phenotype database of genetically modified malaria-parasites. Trends Parasitol.

[5] Li C, Seixas E, Langhorne J (2001). Rodent malarias: the mouse as a model for understanding immune responses and pathology induced by the erythrocytic stages of the parasite. Med Microbiol Immunol.

[6] Stevenson M, Lemieux S, Skamene E (1984). Genetic control of resistance to murine malaria. J Cell Biochem.

[7] Langhorne J, Quin SJ, Sanni LA (2002). Mouse models of blood-stage malaria infections: immune responses and cytokines involved in protection and pathology. Chem Immunol.

[8] Pattaradilokrat S, Culleton RL, Cheesman SJ, Carter R (2009). Gene encoding erythrocyte binding ligand linked to blood stage multiplication rate phenotype in *Plasmodium yoelii yoelii*. Proc Natl Acad Sci USA.

[9] Otsuki H, Kaneko O, Thongkukiatkul A, Tachibana M, Iriko H, Takeo S (2009). Single amino acid substitution in *Plasmodium yoelii* erythrocyte ligand determines its localization and controls parasite virulence. Proc Natl Acad Sci USA.

[10] Li J, Zhang Y, Sullivan M, Hong L, Huang L, Lu F (2007). Typing *Plasmodium yoelii* microsatellites using a simple and affordable fluorescent labeling method. Mol Biochem Parasitol.

[11] Li J, Zhang Y, Liu S, Hong L, Sullivan M, McCutchan TF (2009). Hundreds of microsatellites for genotyping *Plasmodium yoelii* parasites. Mol Biochem Parasitol.

[12] Nair SC, Pattaradilokrat S, Zilversmit MM, Dommer J, Nagarajan V, Stephens MT (2014). Genome-wide polymorphisms and development of a microarray platform to detect genetic variations in *Plasmodium yoelii*. Mol Biochem Parasitol.

[13] Li J, Pattaradilokrat S, Zhu F, Jiang H, Liu S, Hong L (2011). Linkage maps from multiple genetic crosses and loci linked to growth-related virulent phenotype in *Plasmodium yoelii*. Proc Natl Acad Sci USA.

[14] Pattaradilokrat S, Li J, Wu J, Qi Y, Eastman RT, Zilversmit M (2014). *Plasmodium* genetic loci linked to host cytokine and chemokine responses. Genes Immun.

[15] Pattaradilokrat S, Cheesman SJ, Carter R (2008). Congenicity and genetic polymorphism in cloned lines derived from a single isolate of a rodent malaria parasite. Mol Biochem Parasitol.

[16] Wu J, Tian L, Yu X, Pattaradilokrat S, Li J, Wang M (2014). Strain-specific innate immune signaling pathways determine malaria parasitemia dynamics and host mortality. Proc Natl Acad Sci USA.

[17] Carlton JM, Angiuoli SV, Suh BB, Kooij TW, Pertea M, Silva JC (2002). Genome sequence and comparative analysis of the model rodent malaria parasite *Plasmodium yoelii yoelii*. Nature.

[18] Lindner SE, Mikolajczak SA, Vaughan AM, Moon W, Joyce BR, Sullivan WJ (2013). Perturbations of Plasmodium Puf2 expression and RNA-seq of Puf2-deficient sporozoites reveal a critical role in maintaining RNA homeostasis and parasite transmissibility. Cell Microbiol.

[19] Otto TD, Bohme U, Jackson AP, Hunt M, Franke-Fayard B, Hoeijmakers WA (2014). A comprehensive evaluation of rodent malaria parasite genomes and gene expression. BMC Biol.

[20] Lu F, Jiang H, Ding J, Mu J, Valenzuela JG, Ribeiro JM (2007). cDNA sequences reveal considerable gene prediction inaccuracy in the *Plasmodium falciparum* genome. BMC Genom.

[21] Lopez-Barragan MJ, Lemieux J, Quinones M, Williamson KC, Molina-Cruz A, Cui K (2011). Directional gene expression and antisense transcripts in sexual and asexual stages of *Plasmodium falciparum*. BMC Genom.

[22] Watanabe J, Wakaguri H, Sasaki M, Suzuki Y, Sugano S (2007). Comparasite: a database for comparative study of transcriptomes of parasites defined by full-length cDNAs. Nucleic Acids Res.

[23] Otto TD, Wilinski D, Assefa S, Keane TM, Sarry LR, Bohme U (2010). New insights into the blood-stage transcriptome of *Plasmodium falciparum* using RNA-Seq. Mol Microbiol.

[24] Sorber K, Dimon MT, Derisi JL (2011). RNA-Seq analysis of splicing in *Plasmodium falciparum* uncovers new splice junctions, alternative splicing and splicing of antisense transcripts. Nucleic Acids Res.

[25] Bartfai R, Hoeijmakers WA, Salcedo-Amaya AM, Smits AH, Janssen-Megens E, Kaan A (2010). H2A.Z demarcates intergenic regions of the *Plasmodium falciparum* epigenome that are dynamically marked by H3K9ac and H3K4me3. PLoS Pathog.

[26] MR4: https://www.beiresources.org. Accessed 15 Jan 2016.

[27] Peters W, Robinson BL, Mittelholzer ML, Crevoisier C, Sturchler D (1995). The chemotherapy of rodent malaria. LII. Response of *Plasmodium yoelii* ssp. NS to mefloquine and its enantiomers. Ann Trop Med Parasitol.

[28] Tao ZY, Xia H, Cao J, Gao Q (2011). Development and evaluation of a prototype non-woven fabric filter for purification of malaria-infected blood. Malar J.

[29] Read data: accession number SRP066796 http://www.ncbi.nlm.nih.gov/sra/?term=SRP066796.

[30] Sanger website: YM genome sequence http://www.sanger.ac.uk/resources/downloads/protozoa/plasmodium-yoelii.html.

[31] Langmead B, Trapnell C, Pop M, Salzberg SL (2009). Ultrafast and memory-efficient alignment of short DNA sequences to the human genome. Genome Biol.

[32] Langmead B, Salzberg SL (2012). Fast gapped-read alignment with Bowtie 2. Nat Methods.

[33] Trapnell C, Pachter L, Salzberg SL (2009). TopHat: discovering splice junctions with RNA-Seq. Bioinformatics.

[34] Trapnell C, Roberts A, Goff L, Pertea G, Kim D, Kelley DR (2012). Differential gene and transcript expression analysis of RNA-seq experiments with TopHat and Cufflinks. Nat Protoc.

[35] Mortazavi A, Williams BA, McCue K, Schaeffer L, Wold B (2008). Mapping and quantifying mammalian transcriptomes by RNA-Seq. Nat Methods.

[36] Anders S, Huber W (2010). Differential expression analysis for sequence count data. Genome Biol.

[37] Trapnell C, Williams BA, Pertea G, Mortazavi A, Kwan G, van Baren MJ (2010). Transcript assembly and quantification by RNA-Seq reveals unannotated transcripts and isoform switching during cell differentiation. Nat Biotechnol.

[38] Florea L, Song L, Salzberg SL: Thousands of exon skipping events differentiate among splicing patterns in sixteen human tissues. F1000Res 2013; 2:188. 10.12688/f1000research.2-188.v1 PMC3892928 24555089

[39] IGV website : https://www.broadinstitute.org/igv/. Accessed 15 Jan 2016.

[40] TransDecoder: http://transdecoder.sourceforge.net. Accessed 15 Jan 2016.

[41] Li H, Handsaker B, Wysoker A, Fennell T, Ruan J, Homer N (2009). The sequence alignment/Map format and SAMtools. Bioinformatics.

[42] GATK2: http://www.broadinstitute.org/gatk/. Accessed 15 Jan 2016.

[43] Carlton J, Silva J, Hall N (2005). The genome of model malaria parasites, and comparative genomics. Curr Issues Mol Biol.

[44] Katayama S, Tomaru Y, Kasukawa T, Waki K, Nakanishi M, Nakamura M (2005). Antisense transcription in the mammalian transcriptome. Science.

[45] Bicknell AA, Cenik C, Chua HN, Roth FP, Moore MJ (2012). Introns in UTRs: why we should stop ignoring them. BioEssays.

[46] Cenik C, Derti A, Mellor JC, Berriz GF, Roth FP (2010). Genome-wide functional analysis of human 5′ untranslated region introns. Genome Biol.

[47] Chung BY, Simons C, Firth AE, Brown CM, Hellens RP (2006). Effect of 5′ UTR introns on gene expression in *Arabidopsis thaliana*. BMC Genom.

