# Supplementary figures and images for: UTR introns, antisense RNA and differentially spliced transcripts between Plasmodium yoelii subspecies
Source: Malar J. 2016 Jan 20;15:30. doi: 10.1186/s12936-015-1081-9 (PMC4721144; doi:10.1186/s12936-015-1081-9)

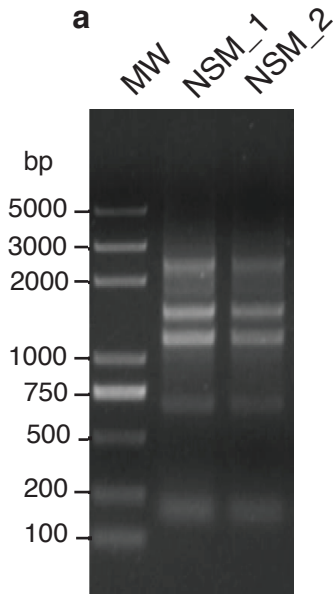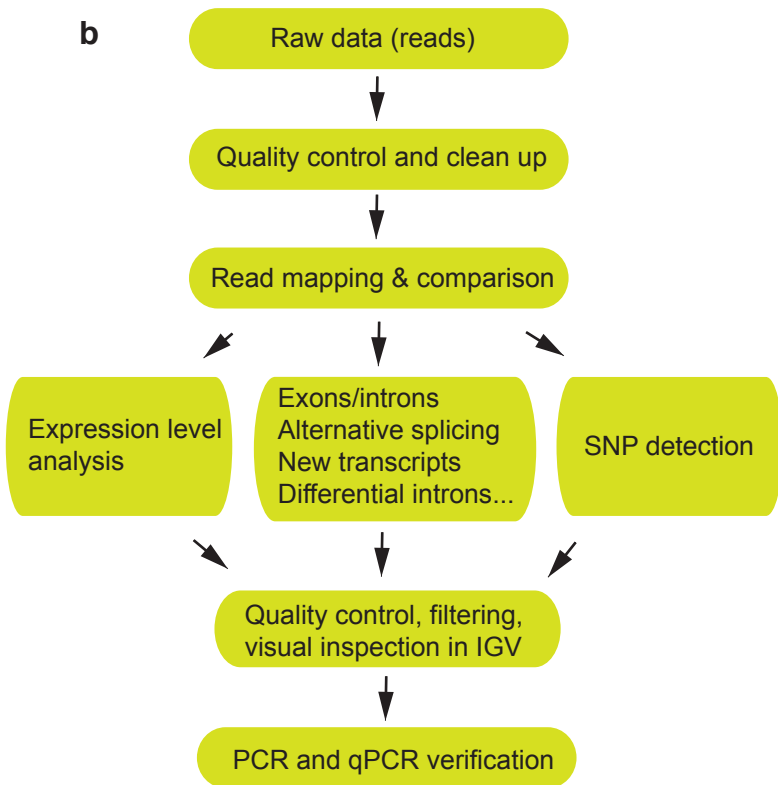

Supplement: Supplementary file 1 — 10.1186/s12936-015-1081-9 Evaluation of RNA quality from the two NSM parasite samples in agarose gel (a), and a flow chart of data processing and analysis (b). [file 12936_2015_1081_MOESM1_ESM.pdf]

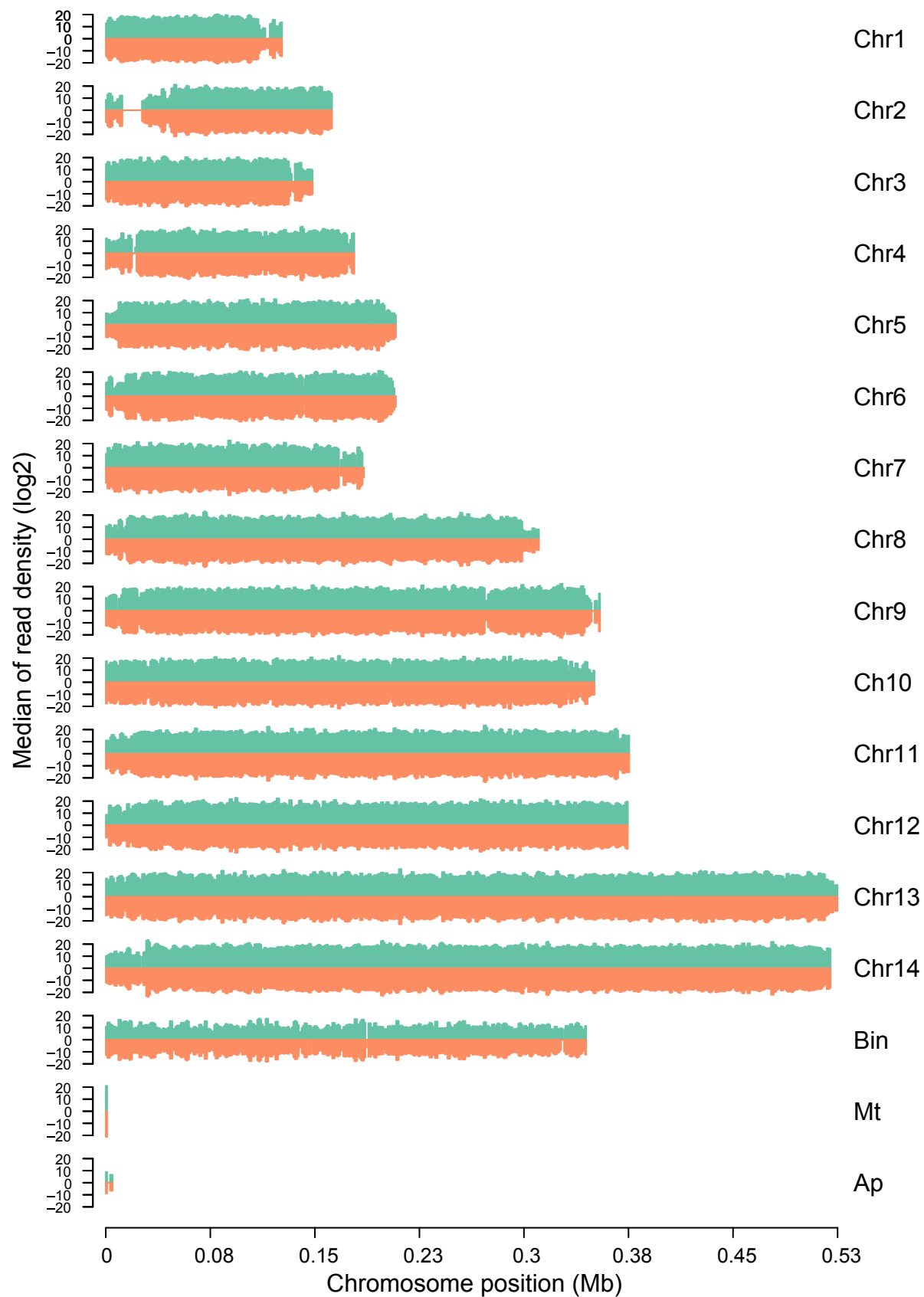

Supplement: Supplementary file 3 — 10.1186/s12936-015-1081-9 Distribution of mapped sequence reads per kb across the 14 parasite chromosomes, mitochondrial (Mt), and apicoplast (Ap) genomes. The green and orange colors indicate ‘+’ and ‘-’ strand coverage, respectively. The chromosomes are as labeled. Only data from NSM_1 are shown. Bin contains sequences not assigned to chromosomes, likely due to gaps in the current assembled genome. [file 12936_2015_1081_MOESM3_ESM.pdf]

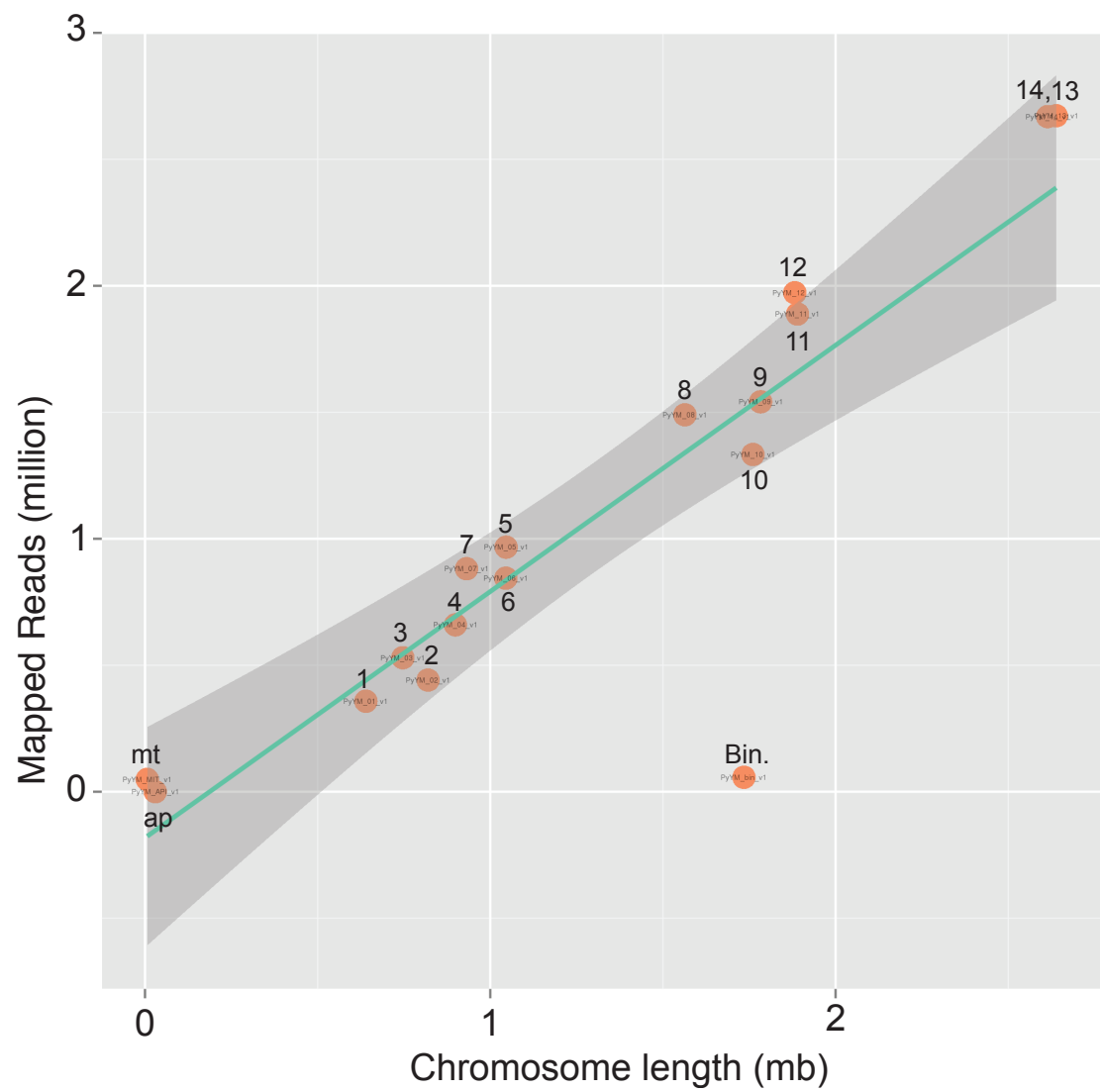

Supplement: Supplementary file 4 — 10.1186/s12936-015-1081-9 Correlation of chromosome length in megabase (Mb) and the numbers of mapped reads. The chromosomes are as labeled. Only data from NSM_1 are shown. The numbers above or below the orange dots indicate chromosomes; Mt, mitochondrial genome; Ap, apicoplast genome; bin, unassigned sequences. [file 12936_2015_1081_MOESM4_ESM.pdf]
